# The Relationship Between Personal Wellbeing, Choice and NDIS Individualised Planning and Support for People With Intellectual Disabilities

**DOI:** 10.1111/jar.70085

**Published:** 2025-07-10

**Authors:** Vivienne Riches, Trevor Parmenter, Gisselle Gallego, Ziad Al‐Rubaie, Mary‐Ann O'Donovan, Patricia O'Brien

**Affiliations:** ^1^ Centre for Disability Studies (CDS), faculty of Medicine and Health Affiliate of the University of Sydney Sydney Australia

**Keywords:** choice, individualised funding, intellectual disability, NDIS, personal wellbeing, Sydney

## Abstract

**Background:**

Australia's National Disability Insurance Scheme (NDIS) funds individualised supports to increase choice and control. The relationship between NDIS individualised funding, outcomes for wellbeing and exercising choice and control for people with intellectual disability has been unclear.

**Method:**

Adult NDIS participants with intellectual disability (*N* = 62) completed a longitudinal survey with validated instruments exploring personal wellbeing and choice.

**Findings:**

Personal wellbeing scores were generally positive, with the mean comparable to that found for a similar population over a decade ago. High choice and control were evident for most individuals over everyday matters, but not key life decisions. Living environment and physical and/or mental and emotional health status were associated with the level of satisfaction with personal wellbeing and everyday choice and control.

**Conclusions:**

There is need to better support people with intellectual disability to exercise choice and control over key life decisions and to address disparities in choice and wellbeing associated with living environment, physical and mental and emotional health and future security.

**Summary**

- Many adult NDIS participants with intellectual disability self‐reported good personal wellbeing. Lowest satisfaction was found in the domains of health and future security.
- Different types and levels of choice and control were being exercised across living environments, with high scores on everyday matters but not key life decisions.
- Personal wellbeing and choice and control were negatively impacted by poor physical and/or mental emotional health.
- Key areas for targeted support and resource allocation were identified.

## 1 Introduction

The Australian National Disability Insurance Scheme Act (NDIS Act) (Commonwealth of Australia 2013), in conjunction with other laws, gives effect to Australia's obligations under the Convention on the Rights of Persons with Disabilities (CRPD) that are to ‘promote, protect and ensure the full and equal enjoyment of all human rights and fundamental freedoms by all persons with disabilities, and to promote respect for their inherent dignity’ (United Nations 2006, Article 1).

The NDIS Act provides for the National Disability Insurance Scheme (NDIS) to support the independence and social and economic participation of people with disability; and to provide funding to eligible people with intellectual and other disabilities so they can access the supports and services they need to live a quality life in their community (NDIS 2021). As of December 2024, NDIS scheme participants with intellectual disability numbered 93,835/692,823 or14% of total participants (NDIS 2024).

Individualised funding packages are developed for eligible NDIS participants based on the creation of individual support plans that identify current and future goals, and the supports considered reasonable and necessary to pursue these goals and lead a life of quality. Once the plan has been approved by the National Disability Insurance Agency (NDIA), a personal budget (PB) is allocated to cover the costs of approved supports (NDIS 2021).

## 2 Choice and Self‐Determination

The right of choice and the ability to have control and engage as equal partners in the decision‐making process are essential features of the NDIS planning process as stated in the NDIS Act (2013; Commonwealth of Australia 2013, S4.8). This aligns with Article 3 of the UN Convention on the Rights of Persons with Disabilities (UNCRPD) principles of *Respect for inherent dignity, individual autonomy including the freedom to make one's own choices, and independence of persons* (United Nations 2006). Nevertheless, the extent to which people with intellectual disability exercise true choice and control in their NDIS planning experiences is unknown. Choice is seen as ‘making an unforced selection of a preferred alternative from two or more options’ (Stancliffe 2001, 92); involves two critical criteria, the opportunity to choose and the act of decision making (Brown and Brown 2009), and is ‘…the experience of autonomy in both small everyday matters and in large, life‐defining matters’ (O’Brien 1987, 177). Furthermore, choice and consumer decision making about services and daily life are central elements of self‐determination (O'Donovan et al. 2017; Wehmeyer 2002; Wehmeyer and Abery 2013). Self‐advocates have been increasingly vocal in identifying the importance of self‐determination in their lives (Abery et al. 2019; Smith et al. 2020).

Fyson and Cromby (2013) noted there is significant variability in the capacity of people with disabilities to make choice decisions, while others have argued that restrictions in life choices and the diminished cognitive capacity of people with intellectual disability may limit their agency for future planning and making meaningful choices (Schelly 2008; Smith 2013). An additional complexity is the controlled environments many people with intellectual disability are often subjected to, especially throughout their early years (Burton‐Smith et al. 2005; Stancliffe et al. 2011). Support system factors also present barriers to people's opportunities to make choices (Stancliffe 2001; Stancliffe et al. 2020; Walker et al. 2011).

Studies have examined the relationship between place of living and the opportunities for people with intellectual disability to exercise choice. Key factors identified which facilitated or restricted opportunities for choice making included the level to which professionals make assessment of the autonomy of the person (Pallisera et al. 2021); type of residence (Cocks et al. 2017; O'Donovan et al. 2017; Stancliffe 2002; Stancliffe et al. 2011); having friends other than co‐residents (O'Donovan et al. 2020) and control over the selection of support staff and rostering (Bigby et al. 2017).

Schalock and Verdugo (2002, 2012) identified self‐determination as a core dimension of quality of life. While consumer choice and self‐determination constitute a key intervention policy and practice concept in the support of people with intellectual disability, there is relatively little quantitative research that has focussed on the relationship between choice, self‐determination and quality of life (Neilly‐Barnes et al. 2008).

### 2.1 Quality of Life and Wellbeing

Quality of life embodies two domains, one objective and one subjective. Both subjective and objective QOL assessments have become popular as a disability program outcome measure for areas including supported community living (Bigby et al. 2017; Bigby and Beadle‐Brown 2018; Cocks, Thoresen, O’Brien 1987; Felce et al. 2008; Parmenter et al. 1990); employment (Beyer et al. 2016; Roessler et al. 1990; Robertson et al. 2019; Scuccimarra and Speece 1990); family (Edwards et al. 2018); and monitoring the fulfilment of the rights set out in several Articles of the UNCRPD at both macro and individual levels (Gómez Sánchez et al. 2022; Lombardi et al. 2019; Schalock and Verdugo 2002).

Wellbeing is considered one subjective indicator of quality of life (Schalock and Felce 2004). Further, the assessment of flourishing or a sense of wellbeing is important and involves more than just life satisfaction and happiness, according to Huppert and So (2013). Cummins (2005) argued that the Personal Wellbeing Index: Intellectual Disability (PWI‐ID) captures the essence of a person's subjective wellbeing (SWB) using seven items, that enables a testable theoretical model of QOL. He further demonstrated that of these seven items, three represent the ‘Golden Domain Triangle’ satisfaction with money, relationships and life achievements. He suggested that *For service provision to be maximally effective in facilitating life quality, it should target sufficiency in these areas* (Cummins 2020, 182). The latest edition of the Personal Wellbeing Index: Adult asserted that for subjective life quality to be measured validly and empirically it is necessary to ask the individual how they feel about aspects of their life (International Wellbeing Group 2024).

At a theoretical level, models of quality of life have embedded choice as a component of quality of life (Brown and Brown 2009; Schalock et al. 2016; Gómez Sánchez et al. 2021). However, Cummins (2005) saw choice as a causal rather than an indicator or outcome variable. He argued that quality of life cannot necessarily be improved by altering the level of choice, because causal variables are in constant interaction with each other. As a way forward he proposed that choice is better placed within the broader construct of control as it is in terms of life quality, a more powerful construct, because it can deliver empowerment, whereas choice is generally limited.

Williams and Porter (2017) pointed out that the United Kingdom 2014 *Care Act* (Care Act, 2014) proposed that the notion of control over everyday life was one element of ‘wellbeing’ which the Act saw as the primary goal of providing social care. An interesting question is the relationship between choice and control and wellbeing, especially whether there is a causal relationship. In other words, does the exercise of choice and control result in higher levels of wellbeing?

Given the dearth of research involving people with intellectual disability, further investigation of the relationship between choice, control and wellbeing is warranted. This paper examines the extent to which Australia's NDIS funding has impacted specifically upon the wellbeing and choice and control of a sample of people with intellectual disability. It also examines potential relationships between the measures of wellbeing and choice. Particularly it asked:

1. To what extent was choice exercised by people with intellectual disability in their obtained support from the NDIS program?
2. To what extent has the NDIS support provisions impacted upon the wellbeing of people with intellectual disability?
3. Is there a relationship between the opportunities for choice‐making and wellbeing?

## 3 Method

This study was part of a larger multiphase mixed methods research project that aimed to examine the relationship between self‐directed individualised funding and its effect on personal wellbeing, self‐esteem and voice, choice and control for people with intellectual disability. All data were deidentified, and the study was approved by the (Sydney) Human Research Ethics Committee (Approval number: 2021/047).

### 3.1 Participants

Participant inclusion criteria comprised *n* = 62 adults aged 18 years and over who have a mild to moderate intellectual disability and are NDIS participants or registered with the NDIA (Table 1). Recruitment was undertaken by eight partner organisation liaison officers. Consent was obtained for participation in an interview and for access to the participant's NDIS plan.

**TABLE 1:** Demographics Wave 1 and Wave 2.

Categorical variables	Wave 1			Wave 2			Wilcoxon Signed Ranks test
	*N*	Percent	Cum %	*N*	Percent	Cum %	
Age total	62	100.0		45	100.0		*Z* = −0.38
17–29 years	13	21.0	21.0	10	22.2	22.2	*p* = 0.71
30–39 years	3	4.8	25.8	1	2.2	24.4	
40–49 years	15	24.2	50.0	9	20.0	40.4	
≥ 50 years	31	50.0	100.0	25	55.6	100.0	
Mean age (SD)	47.6 years (SD = 15.6)			49.0 (16.0)			
Gender	62	100.0		45	100.0		*Z* = −0.15
Male	29	46.8	46.8	22	48.9	48.9	*p* = 0.88
Female	32	51.6	98.4	22	48.9	98.8	
Non/binary 3rd gender	1	1.6	100.0	1	2.2	100.0	
Relationship status	61	100.0		45	100.0		Z = 0.18
Single	58	95.1	95.1	40	88.9	88.9	*p = 0.03
Other	3	4.9	100.0	5	11.1	100.0	
CALD background	61	100.0		44	100.0		Z = −0.30
Yes	7	11.5	11.5	6	13.6	13.6	*p* = 0.76
No	54	88.5	100.0	38	86.4	100.0	
Aboriginal or Torres St Islander	62	100.0		45	100.0		
Yes	0	0	0	0	0.0	0.0	
No	62	100.0	100.0	45	100.0	100.0	
Highest education completed	60	100.0		45			Z = −3.41
Compulsory education—secondary	32	53.3	53.3	35	77.8	77.8	*p = . < 0.001
College/University	27	45.0	98.3	9	20.0	97.8	
Never attended	1	1.7	100.0	1	2.2	100.0	
Employment status	60	100.0		45	100.0		Z = −1.260
Working (full time/part time)	28	46.7	46.7	14	31.1	31.1	*p* = 0.21
Unemployed	11	18.3	65.0	18	40.0	70.1	
Retired	11	18.3	83.3	4	8.9	80.0	
Unpaid voluntary work/family business	5	8.3	91.6	6	13.3	93.3	
Other	5	8.3	100.0	3	6.7	100.0	
Living environment	60	100.0		45			Z = −3.12
Independent (own home) /SIL	30	50.0	50.0	24	53.3	53.3	* p ≤ 0.002
Family home (with family)	13	21.7	71.7	8	17.8	71.1	
Group home	17	28.3	100.0	13	28.9	100.0	
Housing situation	61	100.0					
I or family rent—private landlord	5	8.2	8.2				
Rent (social/community or public)	41	67.2	75.4				
I or family own home	15	24.6	100.0				
In general‐describe health	61	100.0		45	100.0		Z = −1.39
Excellent/very good	23	37.7	37.7	15	33.3	33.3	*p* = 0.16
Good	25	41.0	78.7	22	48.9	82.2	
Fair/poor	13	21.3	100.0	8	17.8	100.0	
Mental/emotional health	61	100.0		45	100.0		Z = ‐2.25
Excellent/very good	13	21.03	21.3	15	34.1	34.1	*p = 0.025
Good	33	54.1	75.4	17	38.6	72.7	
Fair/poor	15	24.6	100.0	12	27.3	100.0	

### 3.2 Study Design and Procedure

A cross‐sectional survey design was used that involved six data sections: participant characteristics, interview data, identity confirmation, personal wellbeing index intellectual disability version (PWI‐ID) (3rd edition, Cummins and Lau 2005), the choice questionnaire (Stancliffe and Parmenter 1999) and questions on NDIS planning, services and supports.

A data protocol was developed and trialled with a convenience sample of adults with intellectual disability before general testing began. This was to ensure questions in the semi‐structured interview could be clearly understood, were meaningful to respondents, and the length of the interview did not cause undue fatigue, including screening for the PWI‐ID.

Interviews were conducted face‐to‐face in an appropriate setting, that is, quiet, comfortable, free from undue distractions and some participants were supported by a support person. A second wave of data was collected a minimum of 12 months after the first wave of data. Participating organisation liaison officers were again used to contact their original participants to gain consent for a second interview involving a repeat of the Choice Questionnaire and the Wellbeing Index plus three questions about their last NDIS planning meeting.

### 3.3 Measures

The Personal Wellbeing Index (PWI‐ID) (3rd edition, Cummins and Lau 2005) measures subjective wellbeing as an indicator of quality of life by rating satisfaction across seven life domains: standard of living, health, life achievement, personal relationships, personal safety, feeling part of the community and future security. The PWI‐ID is a parallel version of The Personal Wellbeing Index (PWI) used in the general population (Cummins et al. 2002; Lau et al. 2005; Tiliouine et al. 2009) designed for people who have intellectual disability or other forms of cognitive impairment. Participants rate how happy they feel (instead of satisfaction) on each of the seven items using visual faces and an 11‐point ruler scale from zero (very sad) through 5 (neither happy nor sad) to 10 (very happy). Raw scores are then converted into ‘percentage of scale maximum’ or % SM on a 0–100 distribution. Scale items may be used at the level of individual domains, and domain scores can be aggregated and averaged to form PWI‐ID (Cummins and Lau 2005, 26–27). The PWI‐ID demonstrated adequate psychometric properties including validity, internal reliability and good test–retest reliability for a sample of 114 individuals with mild to moderate intellectual disability (McGillivray et al. 2009).

The Choice Questionnaire (Stancliffe and Parmenter 1999) is a 26‐item scale that assesses the degree of choice exercised by a person with intellectual disability. It was used for self‐reports (not proxy reports) and reduces acquiescent responses using mainly open‐ended questions. Topics cover choice regarding domestic activities, staff and other people, money and spending, health, social activities, community access and personal relationships, work/day activities and overall choice. A 3‐point scale is used with clear criteria for scoring each item. Total Choice Score can range from 0 to 78. Authors report the scale's wide range of item difficulty makes it useful with individuals who exercise widely differing levels of choice. High internal consistency, concurrent and construct validity, interscorer agreement and sound test–retest reliability for staff reports have been reported (Stancliffe and Parmenter 1999).

### 3.4 Statistical Analysis

The distribution of continuous variables including age, duration of interview, Choice Score Total and PWI‐ID Total Score was inspected visually, and descriptive statistics are reported. The Shapiro–Wilk test assessed the normality distribution of continuous variables using a *p* < 0.05 when the sample was a non‐normal distribution.

Categorical variables are presented as numbers and percentages. The non‐parametric Kruskal–Wallis test was used to test for significant differences in Choice Total and PWI‐ID Total Scores between groups on demographic factors (*p* value reported) for Wave 1 (W1) and Wave 2 (W2) data. Boxplot graphs were visually used to compare Total Score distributions across demographic categories. The association between each choice item, participant characteristics and PWI‐ID Total Score was assessed using Poisson Log Linear Regression.

Choice Questionnaire and PWI‐ID Total Scores were categorised into two categories: High (above the median score) and Low (equal or below median score). The association between each Choice item, participant characteristics and the PWI‐ID Total Score for ‘low’ and ‘high’ Total personal wellbeing were assessed using Univariate and Multivariate Logistic Regression Models (adjusting for age and gender). For rational (robust) findings, some of the categorical variables were re‐categorised and reduced by merging their relevant groups. These analyses were repeated for Wave 2 data.

The non‐parametric Wilcoxon Signed Ranks Test was used to assess any changes in both the Choice Total and PWI‐ID Total between Wave 1 and Wave 2, and the *p* value reported. Mean differences were also assessed across both total scores between subgroups of the demographic factors. Missing data were excluded from the analysis for each variable. The SPSS software package version 29 was used for data analysis.

## 4 Results

### 4.1 Participants

Wave 1 data comprised 62 consenting participants with mild to moderate intellectual disability. Ages ranged from 19 to 70 years, with the average being 47.6 years (SD = 15.6) and median 50.5 years (IQR 35–60 years). There were 29 males (47%), 32 females (52%) and one non‐binary third gender. Only seven of the participants (11.5%) came from a culturally and linguistically diverse (CALD) background. Most participants were single (95%) and 50% were living independently or in supported independent living (SIL) situations. Another 21.7% lived with family in the family home, and the remaining 28% lived in group home settings. Three quarters of the sample lived in rental accommodation, while a quarter were in a personally or family‐owned home. Most participants indicated a preference for living in their own home.

Over half the sample had completed secondary education and 45% had attended some form of postsecondary college or university audited programme. Twenty‐eight were employed full or part time (47%), five were doing unpaid voluntary work (8.3%), others were retired (18.3%), unemployed (18%) or in other situations, for example, day programme (8.3%). Demographic details are reported in Table 1.

A total of 45 participants were re‐interviewed at Wave 2. Wilcoxon Signed Ranks Tests revealed there were few significant differences in personal characteristics between the participants interviewed at Wave 1 and Wave 2 (see Table 1). However, a significantly greater proportion of W1 participants were single (*p* = 0.03), had completed some form of post‐secondary education (*p* = < 0.001), were living in the family home (*p* = < 0.002) and rated their mental emotional health as excellent or very good (Table 1).

**FIGURE 1:**
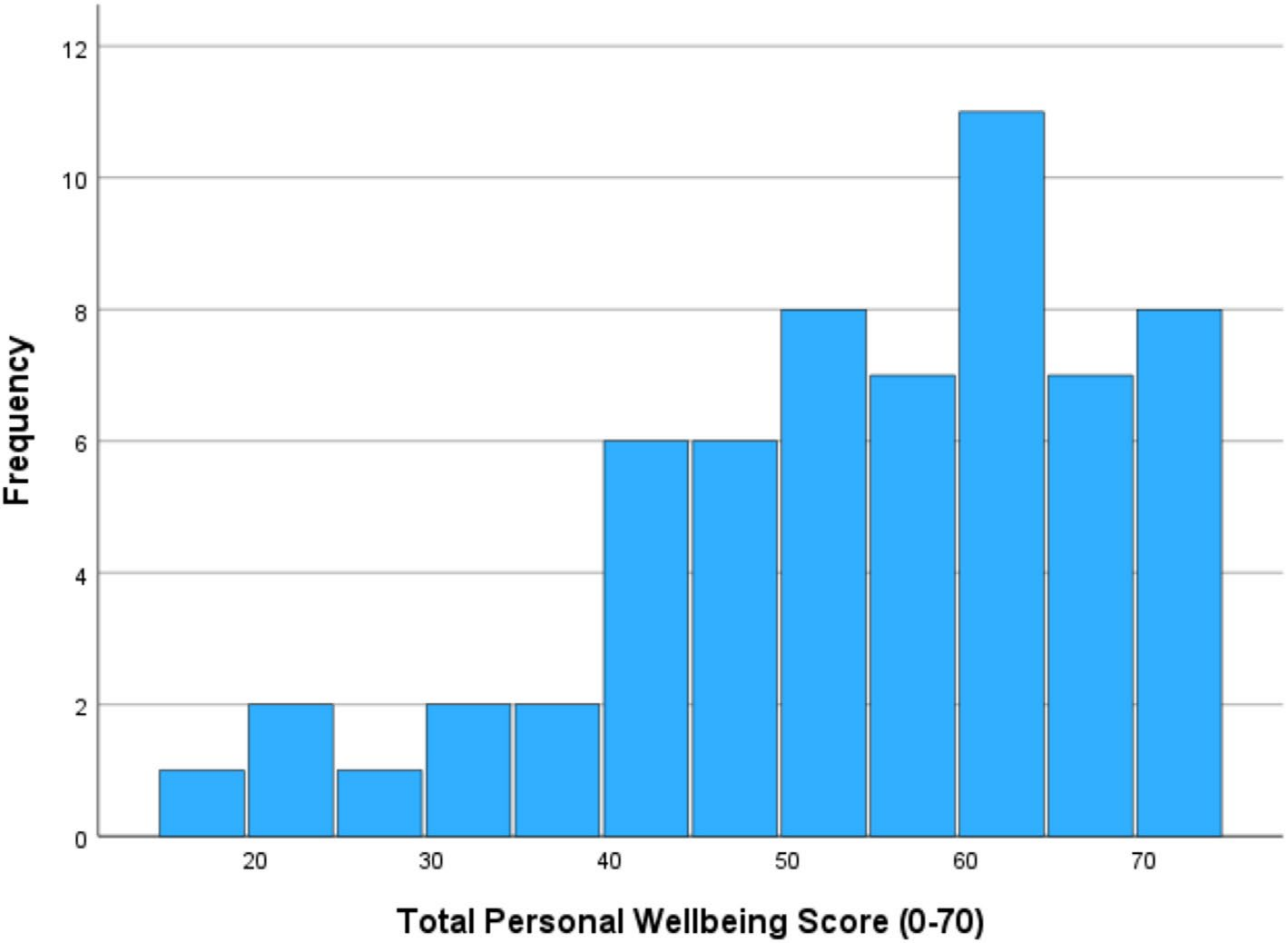
Distribution for personal wellbeing total raw scores Wave 1.

Total Scores on the PWI‐ID revealed a positively skewed non‐normal distribution (*p* < 0.001) with percentage scores ranging from 24.3 to 100. Several participants scored very poorly (Figure 1). The PWI‐ID mean at wave 1 was *M* = 76.57 (SD =21.70) and *M* = 79.70 (SD = 19.0) at Wave 2. No significant differences were evident between Wave 1 and Wave 2 PWI‐ID scores with *p* = 0.26 (Table 2). This level of satisfaction was comparable with the results (PWI‐ID = 77.1, SD = 16.64) reported for a similar population of people with mild to moderate intellectual disability (McGillivray et al. 2009) and slightly above the normative range of wellbeing according to the PWI for the Australian population consistent over 16 years at *M* = 74.5 (SD = 12.5, range 74.2–76.8) reported by Capic et al. (2018).

**TABLE 2:** Items on Personal Wellbeing Index‐ ID Wave 1 and 2.

PWI‐ID domain	Wave 1 (*N* = 62)		Wave 2 (*N* = 45)	McGillivay (2009) (*N* = 77)		
	Mean	SD	Mean	SD	Mean	SD
How happy do you feel about…? – The things you have, like the money you have and the things you own? (Standard of living)	83.61	28.0	79.78	26.50	75.18	26.86
2 How happy do you feel about…?—How healthy you are? (Health)	70.49	31.6	74.00	31.36	70.49	26.39
3 How happy do you feel about…?—The things you make or the things you learn (Life achievement)	81.48	24.0	83.80	20.37	79.30	26.05
4 How happy do you feel about…?—Getting on with the people you know? (Personal relationships)	78.20	30.4	81.56	22.96	82.06	24.19
5 How happy do you feel about…?—How safe you feel? (Personal safety)	73.93	32.2	74.30	27.90	79.25	23.12
6 How happy do you feel about…?—Doing things outside your home? (Feeling part of the community)	78.20	29.7	76.40	24.97	81.84	23.10
7 How happy do you feel about…?—How things will be later on in your life? (Future security)	69.84	32.0	76.80	27.26	72.41	26.44
PWI‐ID Total Kruskal Wallis W1:W2, *p* = 0.26	76.57	19.56	79.70	19.01	77.08	16.64

Greatest satisfaction across the wellbeing domains was evident in the domain concerning possession of money and things owned (W1 *M* = 83.61, SD = 28.0), followed by satisfaction with things made or learnt (W1 *M* = 81.48, SD = 24.0). There was also satisfaction in getting on with known people (W1 *M* = 78.20, SD = 30.4) and doing things outside the home (*M* = 78.20, SD = 29.7). Lowest mean ratings were on the domains for health (W1 *M* = 70.49, SD = 31.6) and how they think things will be in the future (W1 *M* = 69.84, SD = 32.0). These domain rating scores compared favourably in both size and direction with results reported by McGillivray et al. (2009) where mean domain satisfaction levels ranged from 70.49 through 82.06, with lowest domain ratings also found for health (*M* = 70.49) and future security (*M* = 72.41).

No associations were found between demographics involving age, gender, residential setting and so on, and the PWI‐ID Total. However, at both rating periods, higher PBI‐ID Total Scores were positively associated with higher self‐ratings for both physical health (W1 *p* = 0.003; W2, *p* = 0.001) and mental–emotional health (W1 *p =* 0.004; W2 *p* = 0.001). Scores for individuals who rated their health as excellent or very good health had highest personal wellbeing scores (W1 *M* = 84.86; W2 *M* = 90.14), while significantly lower scores were recorded by participants whose health was rated as ‘fair or poor’ (W1 *M* = 61.28; W2 *M* = 61.57). Significantly higher personal wellbeing scores were also found among individuals who rated their mental and emotional health as ‘excellent/very good’ (W1 *M* = 89.86; W2 *M* = 90.0). Lower scores were recorded by participants whose mental and emotional health was ‘good’ (W1 *M* = 76.14; W2 *M* = 78.57), and lowest scores were recorded for participants whose mental and emotional health was ‘fair/poor’ (W1 *M* = 65.71; W2 *M* = 61.57) (Table 3).

**TABLE 3:** Personal Wellbeing Total and demographics Wave 1 and Wave 2 and Association between Participant Characteristics and Total Personal Wellbeing Score [continuous] using Poisson Log Linear Regression model.

	Personal Wellbeing Index‐ID Total Wave 1				Personal Wellbeing Index‐ID Total Wave 2				Poisson log linear regression			
	*N*	Mean	SD	Kruskal‐Wallis *p* value	*N*	Mean	SD	Kruskal‐Wallis *p* value	RR (95% CI)	*p*	Adjusted RR (95% CI)*	*p*
Age	61	76.6	21.70	0.79	45	77.6	19.0	0.07				
Gender	61	76.6	21.70	0.23	45	77.6	19.0	0.69				
CALD background	60	76.6	21.70	0.32	44	77.6	19.0	0.43				
Relationship status	61	76.6	21.70	0.83	45	77.6	19.0	0.97	0.99 (0.75–1.30)	0.94	0.98 (0.75–1.29)	0.89
Highest education level	60	76.3	21.70	0.31	45	77.6	19.0	0.57	1.05 (0.93–1.18)	0.46	1.06 (0.95–1.19)	0.30
Employment status	60	76.6	19.71	0.56	45	77.6	19.0	0.83	1.02 (0.98–1.07)	0.29	1.02 (0.98–1.06)	0.35
Where live	60	76.3	19.57	0.36	45	77.6	19.0	0.22	1.04 (0.97–1.12)	0.23	1.05 (0.98–1.13)	0.14
Health	61	76.6	19.57	0.003*	45	77.6	19.0	0.001*	0.86 (0.80–0.93)	< 0.001	0.87 (0.79–0.96)	0.005*
Excellent/very good		84.9	12.57		15	90.1	9.3		1.00		1.00	
Good		76.9	20.86		22	76.3	15.7		0.91 (0.80–1.02)	0.11	0.91 (0.81–1.03)	0.13
Poor		61.3	19.29		8	58.0	24.1		0.72 (0.61–0.86)	< 0.001	0.74 (0.60–0.92)	0.006*
Mental/emotional health	61	76.6	19.57	0.004*	44	77.9	19.1	0.001*	0.86 (0.79–0.92)	< 0.001	0.87 (0.79–0.96)	0.004*
Excellent/very good		89.9	20.14		15	90.0	9.3		1.00		1.00	
Good		76.1	19.71		17	78.6	15.4		0.85 (0.77–0.94)	0.001	0.86 (0.78–0.95)	0.003*
Fair/poor		65.7	7.14		12	61.6	22.1		0.73 (0.63–0.86)	< 0.001	0.76 (0.62–0.92)	0.006*

*Note:* * *p* > 0.05.

Few participants [P] who recorded high PWI‐ID scores commented on their NDIS planning experience, although those who did were mostly satisfied. One participant with PWI‐ID = 100 said ‘I like to go learn painting. I put down I want to learn about computers and woodwork and that happened, but when there was COVID we couldn't do it. … I learned to use a ride‐on‐lawnmower’ [P#7]. Another participant with a PWI‐ID = 92.86 commented ‘They've (NDIS) never said that I can't do more arts and craft. Everything that I do is what I want to do’ (P#43).

In contrast, the lowest scoring participant who had a total PWI‐ID = 24.29 expressed dissatisfaction with their NDIS planning experience ‘I wanted more 1:1 support to see people more often because I feel very anxious a lot of the time, and the person from the NDIS said no—they said I'm too bright too capable I seem to be Ok and I don't need it. That made me feel sad and they need to treat me with more dignity, respect and be a bit more generous. Now I get 1:1 support 3 days a week and 1 day at (workplace). People are nice there. It's part of my service provider. I can get into the productive and fun stuff’ (Participant [P #36]).

Two other low scoring participants (PWI‐ID = 28.57) also made comments about their packages. One stated ‘I was supposed to get a mobility scooter but then I was told I couldn't have one because it couldn't be kept at where I live. I was told it wasn't part of the Plan’ [P#61]. The other explained their dissatisfaction: ‘… My goal right now is to concentrate on my physical health which is in danger. I need based on Drs evidence (thermo regulation) some heating and cooling, but the NDIS have said no’. And ‘They're not listening and they're not reading. We are waiting on the review at the moment. Mobility allowance. Twice a week I work at (workplace). They've taken it away $100 per fortnight but I need that to get to work. I've only been to one NDIS Planning meeting—they assumed I was under (name of service provider) but I wasn't. There are things in my plan I don't recognize. I need the NDIS to keep me independent, like people that will listen to me. People twist things. They do what they want and what they understand’ [P#19].

### 4.2 Choice Questionnaire

**FIGURE 2:**
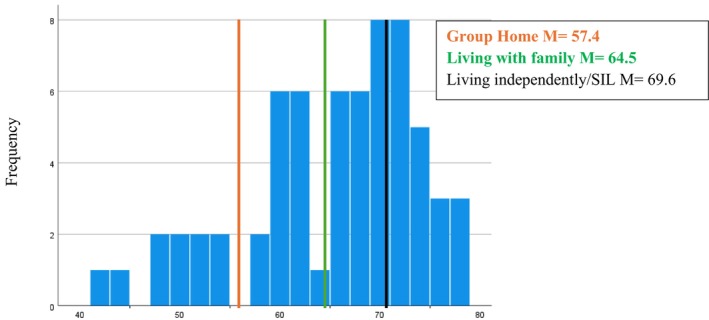
Distribution of choice total scores and living environment at Wave 1.

The Choice Questionnaire Total scores ranged from 42 to 78 at Wave 1 (W1) and the distribution was positively skewed (*p* = 0.006) (Figure 2). The mean was 64.7 (SD = 8.7), and the median was 67. The Wave 2 mean was 64.9 (SD = 10.0) and no significant differences were found between Choice Total scores at W1 and W2 (*p* = 0.92) (Table 4).

**TABLE 4:** Choice Questionnaire Total results and demographics Wave 1 and 2.

	Choice Questionnaire Total Wave 1				Choice Questionnaire Total Wave 2			
	*N*	Mean	SD	Kruskal‐Wallis test *p* value	*N*	Mean	SD	Kruskal‐Wallis test *p* value
Age	62	64.6	8.7	0.70	45	65.0	10.0	0.67
Gender	62	64.6	8.7	0.54	45	65.0	10.0	0.99
CALD background	61	64.5	8.8	0.46	44	64.7	10.0	0.45
Relationship status	62	64.6	8.7	0.97	45	65.0	10.0	0.61
Education level	60	64.8	8.7	0.74	45	65.0	10.0	0.27
Employment status	61	64.6	8.7	0.91	45	65.0	10.0	0.03*
Working full/part time		66.0	8.3			63.9	11.1	
Unemployed		66.0	7.5			69.3	9.6	
Retired		61.4	10.5			54.0	5.7	
Unpaid voluntary work		65.4	4.9			61.5	7.4	
Other		63.8	12.9			65.3	2.1	
Where live	61	64.5	8.8	< 0.001**	45	65.0	10.0	0.03*
Independent		69.5	5.2			67.6	9.9	
Family home		64.5	6.6			67.6	6.9	
Group home		55.9	8.5			58.5	9.5	
Health	61	64.6	8.8	0.28	45	65.0	10.0	0.42
Mental/emotional health	61	64.6	8.8	0.62	44	65.4	9.8	0.54

*Note:* *p < 0.01 **p = 0.001.

Responses to individual choice items were examined using means and percentage ratings. Results are presented in the Table 6 in order of greatest to least mean scores, indicating greatest to least choice and control across the sample at W1.

Just under half the sample (46.8%) strongly agreed that *overall* … *your life is free so you can choose what you want? All the time?* Another 48% responded that they had choice most of the time, although sometimes it was planned for them. A small minority (4.8%) indicated they had little choice and that others decide or mostly decide for them.

Greatest personal choice, independence or control was apparent in everyday matters such as using a phone or mobile without restriction (*M* = 3.0, 96.8%), going out wherever one wanted (*M* = 2.9, 95.2%), getting a drink or snack whenever one wanted (*M* = 2.9, 93.5%), deciding the time to go to bed (*M* = 2.9, 91.9%) and choosing to not/look at sexy (X rated magazines, videos, etc.) (*M* = 2.9, 93.4%). Many participants exercised total choice and control, but others gained or required some support or permission on items such as choosing to exercise or play sport, deciding what to do in spare time, and visiting family or friends (all *M* = 2.8). Next followed a cluster of items around deciding about going to hotels and clubs, deciding who has a key to the house, being able to be in the house alone, choosing to drink or not drink alcohol and choosing which jobs to do around the house (*M* = 2.6).

More participants indicated they had less choice or that decisions were made for them on the items choosing to leave their job or day activity program, choosing to take a day off work, spending money on gambling, determining rules for the house and/or what clothes to buy (*M* = 2.5). Lower mean scores comprised choosing what to cook for dinner (*M* = 2.4), being able to come home late (*M* = 2.3), deciding the amount of money to withdraw from the bank and deciding if they can have a pet (*M* = 2.2). Lowest choice and control items involved budgeting (*M* = 1.8), choosing staff in a group home (*M* = 1.7) and supporting attendance at doctors or dentist appointments (*M* = 1.6).

No significant associations were found between the choice questionnaire total scores and age (*p* = 0.68), gender (*p* = 0.47), relationship status (*p* = 0.63), employment status at W1 (*p* = 0.97), highest level of education (*p* = 0.77), type of housing, that is, rented or owned (*p* = 0.61), health (*p* = 0.20) and mental and emotional health (*p* = 0.59) (Table 4).

**FIGURE 3:**
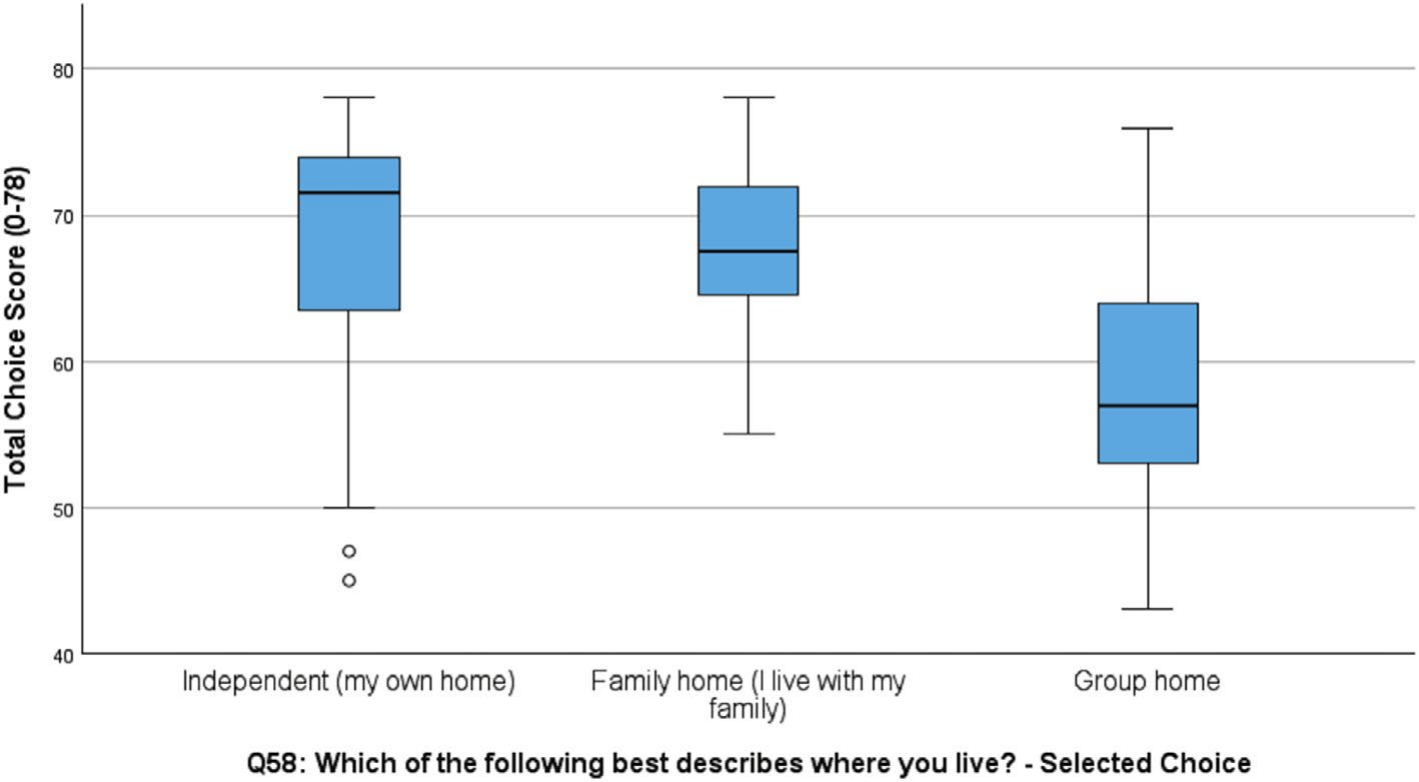
Choice and living arrangement Wave 1.

**FIGURE 4:**
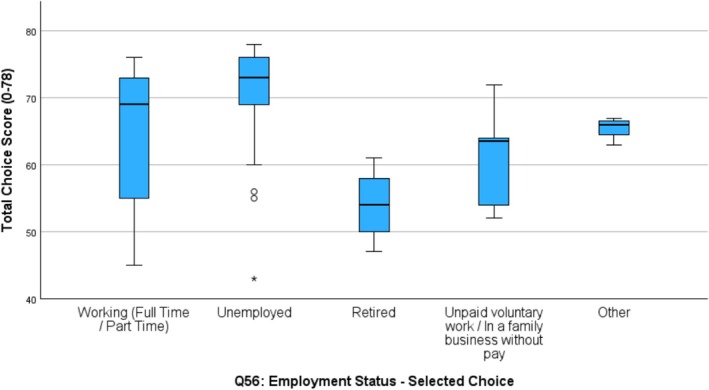
Choice and employment status Wave 1.

However, a significant difference was found in degree of choice respondents had and where they lived (W1 *p* = 0.001; W2 *p* = 0.03). Participants living independently or in supported independent living settings (SIL) had higher total scores indicating greater choice (W1 *M* = 69.6; W2 *M* = 67.6), than those living with family in the family home (W1 *M* = 64.5; W2 *M* = 67.6). Significantly less choice was found among people living in group home settings (W1 *M* = 57.4; W2 *M* = 58.5). A significant association was also found at W2 between the Choice Total score and employment status, with significantly lower choice scores found for participants who had retired (*M* = 54.0). (Table 5 and Figures 3 and 4).

**TABLE 5:** Associations between key participant characteristics, choice questionnaire, and Total Personal Wellbeing score [categorical] using univariate and multivariate logistic regression models, *N* = 61.

Variable	*N* (%)	Total Personal Wellbeing score (17–70)		OR (95% CI)	*p*	Adjusted OR (95% CI)^a^	*p*
		High > 58	Low ≤ 58				
Employment status—Selected choice							
Total	60	27	33				
Working (full/part time)	28 (46.7)	12 (44.4)	16 (48.5)	1.00		1.00	
Unemployed	11 (18.3)	4 (14.8)	7 (21.2)	1.31 (0.31–5.53)	0.71	1.33 (0.29–6.03)	0.71
Retired	11 (18.3)	4 (14.8)	7 (21.2)	1.31 (0.31–5.53)	0.71	1.56 (0.28–8.64)	0.61
Unpaid voluntary	5 (8.3)	3 (11.1)	2 (6.1)	0.50 (0.07–3.48)	0.48	0.60 (0.08–4.70)	0.63
Other	5 (8.3)	4 (14.8)	1 (3.0)	0.19 (0.02–1.90)	0.16	0.18 (0.02–2.04)	0.17
What is the highest year of education you have completed?							
Total	60	26	34				
Compulsory education (secondary education)	32 (53.3)	14 (53.8)	18 (52.9)	1.00		1.00	
TAFE/University (audit)	27 (45.0)	11 (42.3)	16 (47.1)	1.13 (0.04–3.19)	0.82	0.81 (0.24–2.74)	0.74
Never attended	1 (1.7)	1 (3.8)	0 (0.0)	—	—	—	—
In general, how would you describe your—Health							
Total	61	27	34				
Excellent/very good	23 (37.7)	13 (48.1)	10 (29.4)	1.00		1.00	
Good	25 (/)	12 (44.4)	13 (38.2)	1.41 (0.45–4.40)	0.56	1.27 (0.37–4.35)	0.71
Fair/poor	13 (21.3)	2 (7.4)	11 (32.4)	7.15 (1.28–39.83)	0.03	5.43 (0.88–33.47)	0.07
In general, how would you describe your—Mental and Emotional Health							
Total	61	27	34				
Excellent/very good	13 (21.3)	11 (40.7)	2 (5.9)	1.00		1.00	
Good	33 (54.1)	14 (51.9)	19 (55.9)	7.46 (1.42–39.15)	0.02	7.54 (1.33–42.76)	0.02^a^
Fair/poor	15 (24.6)	2 (7.4)	13 (38.2)	35.75 (4.30–297.26)	< 0.001	27.03 (3.04–240.38)	0.003**
Q12: Are there any rules in your house? Who makes up the rules for your house? (Do not include rules imposed in the lease or by the landlord)							
Total	61	27	34				
Others decide the rules. I have no real say.	6 (9.8)	1 (3.7)	5 (14.7)	3.00 (0.32–28.19)	0.34	2.96 (0.26–33.73)	0.38
I/we (the residents) decide the rules with help	15 (24.6)	11 (40.7)	4 (11.8)	0.22 (0.06–0.81)	0.02	0.09 (0.02–0.47)	0.005**
There are no rules (except the landlord's rules in the lease) OR I/we (the from others. residents) decide the rules.	40 (65.6)	15 (55.6)	25 (73.5)	1.00		1.00	
8. Do you have your own key to the house? Do staff have keys to your house? Did you give them the key? Who says which people can have a key?							
Total	61	27					
I do not have a key. I have no real say about who has a key	10 (16.4)	6 (22.2)	4 (11.8)	0.46 (0.11–1.84)	0.27	0.12 (0.02–0.75)	0.02^a^
I have a key, but others mostly decide who else also has a key	2 (3.3)	1 (3.7)	1 (2.9)	0.69 (0.04–11.68)	0.80	0.18 (0.01–3.56)	0.26
I have a key. Staff do not have keys OR I/we (the residents) decide who can have a key. I have a fair say.	49 (80.3)	20 (74.1)	29 (85.3)	1.00		1.00	

^a^ Adjusted for age and gender.

### 4.3 Association Between Choice and Personal Wellbeing

Overall, univariate and multivariate logistic regression models reported in Table 6 revealed there were several significant associations between the Choice Total score and PWI‐ID Total score. Significantly higher choice scores were associated with higher physical health and mental and emotional health scores. The associations between total wellbeing and education and employment items were difficult to interpret and needed further investigation.

**TABLE 6:** Choice Questionnaire Item Results Wave 1 (*N* = 62) (highest to lowest rating).

Choice item	Mean (0–3)	*N*	Percent
Q5: What rules are there about a telephone or mobile phone? Can you use this whenever you want to?	3.0	62	100.0
I am unable or not allowed to use a phone OR my telephone/mobile use is restricted (e.g., only allowed to ring at specified times or to certain places or limited to local calls only)		0	0.0
I can usually ring up. I may ask staff first. There may be minor restrictions (e.g., can't talk for too long if others want to use a phone).		2	3.2
I can ring up without restrictions whenever the phone is not being used. OR I have my own mobile phone		60	96.8
Q18: Does anyone stop you from going out? Is there anywhere you are not allowed to go?	2.9	62	100.0
Others often stop me going out. OR I am not allowed to go to quite a few places.		2	3.2
There are 1 or 2 places I am told not to go to.		1	1.6
No‐one stops me. I can go wherever I want.		59	95.2
Q4: Can you get yourself a drink or something to eat whenever you want? Any time? Do you have to ask someone first?	2.9	62	100.0
No. I am not usually allowed to have snacks and/or drinks OR I can only have them on special occasions.		1	1.6
I can usually have a drink or a snack but I have to ask first.		3	4.8
Yes. I can have a drink or snack whenever I want.		58	93.5
Q22: Does anyone stop you from looking at sexy [X rated] magazines, videos or movies? (If the person says “I don't look at those things,” ask: Who decided that?)	2.9	61	100.0
I am not allowed to. OR I am only allowed to look at things some else says are okay.		3	4.9
Usually no‐one stops me. Occasionally they may ask me not to. OR I decided with help not to.		1	1.6
No. I can look at anything I want (in private). OR I decide not to.		57	93.4
Q1: Who decides what time you go to bed? (Does anyone tell you what time to go to bed? Are there any rules about what time you should go to bed?)	2.9	62	100.0
I have a set bedtime. OR Others mostly tell me when to go to bed.		3	4.8
I usually decide with help. Sometimes others tell me.		2	3.2
I decide for myself.		57	91.9
Q17: Do you do exercise or play sport? Who decides that? (Does anyone make you do exercise or sport?)	2.8	61	100.0
Others mostly decide. OR I am made to do exercise/sport		1	1.6
I usually decide with help.		8	13.1
I decide.		52	85.2
Q19: Who decides what you do in your spare time (when you are not working or at day activities)?	2.8	62	100.0
Others mostly decide.		2	3.2
I usually decide with help.		11	17.7
I decide.		49	79.0
Q21: Who decides if you can go and visit your family and friends [whenever it is all right with them]? Do you ask anyone first? Who?	2.8	61	100.0
Others decide. OR I am not allowed to visit		1	1.6
I can visit but I ask someone (other than the person I am visiting) first.		13	21.3
I can visit whenever it's okay with my family or friends.		47	77.0
Q20: Who decides if you can go to hotels and clubs? (Does anyone try to stop you?	2.6	62	100.0
Others mostly decide. OR I am not allowed to.		6	9.7
I usually decide with help.		14	22.6
I decide.		42	67.7
Q8: Do you have your own key to the house? Do staff have keys to your house? Did you give them the key? Who says which people can have a key?	2.6	62	100.0
I do not have a key. I have no real say about who has a key		11	17.7
I have a key, but others mostly decide who else also has a key		2	3.2
I have a key. Staff do not have keys OR I/we (the residents) decide who can have a key. I have a fair say.		49	79.0
Q10: What rules are there about you being by yourself in the house [by yourself and without staff]? Can you be by yourself in the house if you want to? Anytime? If person lives alone score as 3 (i.e., no restrictions).	2.6	62	100.0
I am not allowed to be by myself. I never am by myself in the house.		8	12.9
Sometimes I can be by myself (e.g., only in certain situations or for short periods 1–2 h).		7	11.3
I can be by myself in the house at any time with no restrictions		47	75.8
Q16: Do you drink alcohol like beer or wine? (Yes/No) Who decided that you do/don't drink beer/wine? (Text) Do you ask anyone if you can drink alcohol? (Yes/No) Who? (If person drinks: Does anyone try to stop you drinking alcohol? If person does not drink	2.6	57	100.0
Others decide (e.g., say I am not allowed to drink).		5	8.8
I decided with help OR I ask someone (staff or family) first OR I don't drink because of the medication I take or other medical reasons OR I drink but there are some restrictions on my drinking.		11	19.3
I decided. I am free to drink or not.		41	71.9
Q2: Who decides which jobs you do around the house? Do you have set jobs or a jobs roster? Who works out the roster/set jobs?	2.6	62	100.0
Others mostly tell me. OR My jobs are set by a jobs roster or list made up by someone else.		4	6.5
I/we help staff make up the jobs roster.		14	22.6
I/we (the residents) choose the jobs I/we do		44	71.0
Q23: Can you leave your job/day activity if you want to do no work and just stay at home? Would you ask anyone first? (Would anyone try to stop you leaving if you wanted to?)	2.5	62	100.0
Others decide OR I am not allowed to leave OR I have never had a job or day activities.		11	17.7
I participate in the decision and discuss it with others.		7	11.3
Yes, I can leave if want to. I don't have to ask anyone else. OR I have already left my job/activity & it was completely my decision.		44	71.0
Q14: Do you spend some money on gambling like lottery tickets, lotto, poker machines or the TAB? Who decides that you do/don't gamble? (Can you gamble if you want to?)	2.5	57	100.0
I am not allowed to gamble.		10	17.5
I usually decide with help. OR Sometimes others may tell me not to.		6	10.5
I decide.		41	71.9
Q7: Are there any rules in your house? Who makes up the rules for your house? (Do not include rules imposed in the lease or by the landlord.)	2.5	62	100.0
Others decide the rules. I have no real say.		7	11.3
I/we (the residents) decide the rules with help		15	24.2
There are no rules (except the landlord's rules in the lease) OR I/we (the from others. residents) decide the rules.		40	64.5
Q25: What happens if you want to take a day off work/day activities when you are not sick? You just feel like having a day off. Do you have to ask anyone first?	2.5	62	100.0
Others decide – I have no real say. OR I am not allowed. I have to go to work/day activities OR I do not make this choice because I never work or attend day activities.		13	21.0
I decide with help. I ask others (e.g., staff or family) first.		8	12.9
It is my decision. (I might lose a day's pay)		41	66.1
Q13: When you buy your clothes who chooses which clothes to buy?	2.5	62	100.0
Others mostly decide OR Others buy clothes for me.		5	8.1
I usually choose my clothes with help (e.g., someone usually goes with me).		23	37.1
I choose. I buy my clothes with no help.		34	54.8
Q3: When you cook dinner, who chooses what you cook? Do you ask the others who live here what they would like to eat?	2.4	62	100.0
I don't cook dinner (or I only help with cooking) OR Others mostly choose what I cook OR There is a planned menu made up by someone else.		11	17.7
I usually choose with help from staff.		16	25.8
I choose (I may check with other residents to see what they do/don't like).		35	56.5
26. Overall, would you say that your life is free so you can choose what you want? All the time?	2.4	62	100.0
No. I often cannot do what I want.		3	4.8
Yes, most of the time. Sometimes it is planned for me.		30	48.4
Yes definitely.		29	46.8
Q24: Can you be late home from work/day activities? Do you have to tell anyone first or ring up? (Do you get into trouble for being home late?)	2.3	62	100.0
Others decide. OR I am not allowed to be late. OR I have no opportunity to stop off after work & get home late because I never go to work/day activities or because I am driven straight home.		13	21.0
I can be late if I want, but I am supposed to ask/tell anyone first or ring up. I get into trouble if I don't tell someone or ring up.		16	25.8
I can come home when‐ ever I like. I don't have to tell someone first or ring up		33	53.2
Q12: Who decides how much money you take out of your bank account? Can you take out as much as you want? Do you ask anyone how much to take out?	2.2	62	100.0
Others mostly decide how much to withdraw, and I am not consulted.		13	21.0
I have help to decide how much to take out OR I ask someone how much to take out OR I have a limit on how much I can withdraw.		23	37.1
I decide without help and with no restrictions on how much to withdraw.		26	41.9
Q9: Who decides if you can have a pet [like a dog, a bird or goldfish] if you want you have any kind of pet you want. Do you have to ask anyone before you get a pet?	2.2	62	100.0
I am not allowed to have a pet. OR Others decide and I have no real say.		13	21.0
There may be some restrictions (e.g., on the type of pet) because of my lease/landlord. OR I have to ask others first.		23	37.1
I can have any pet I like with no restrictions.		26	41.9
Q11: Who works out your budget so you will have enough money?	1.8	62	100.0
Others budget my money and I have little say. OR I have a fixed budget worked out by others.		25	40.3
I have help budgeting my money.		23	37.1
I budget my own money without assistance.		14	22.6
Q6: Who picks the staff to work in your house? (Do you interview new staff to decide who will get the job? Are you asked what you think about new staff?)	1.7	57	100.0
Others choose the staff. I am not consulted and have no real say about who works in my house.		27	47.4
I/we (the residents) participate in choosing staff‐ e.g., I am asked for my views about new staff		19	33.3
I/we (the residents) are responsible for deciding which staff will be employed (e.g., I sit on interview panels).		11	19.3
Q15: Does anyone go with you to see the doctor and the dentist? Who? Does … always go?	1.6	62	100.0
I (almost) always go with staff or family (e.g., parents).		30	48.4
Staff or family (e.g., parents) come with me to some appointments (e.g., specialists).		25	40.3
I always go by myself or with a friend.		7	11.3
Kruskall Wallis	Mean	SD	Asym
Choice Total Time 1	64.7	8.7	Sig (2 tailed)
Choice Total Time 2	64.9	10.0	0.92

Two individual choice items were also significantly related to the PWI‐ID Total. Participants who were supported to make decisions about the rules in the home had higher wellbeing scores than individuals who had less choice (*p* = 0.005). Individuals who reported they had a key to their home also had significantly higher wellbeing scores that those who did not have a key to their home and/or who had no real say on who had a key to the home (*p* = 0.02) (Table 6).

## 5 Discussion

This study explored to what extent choice was exercised by people with intellectual disabilities in their obtained support from the NDIS program; to what extent the NDIS support provisions have impacted upon the wellbeing of people with intellectual disabilities and the relationship between opportunities for choice‐making and wellbeing.

### 5.1 Choice and the NDIS

Different types and levels of choice and control were found among participants with intellectual disabilities now supported under the NDIS scheme. High levels of everyday choice were generally being exercised across the various living environments on everyday matters such as unrestricted use of phones, access to food and drink, community access, visiting family and friends and making use of leisure and sport time.

Participants living independently with or without family support and those in supported living accommodation had significantly higher levels of choice and control, while less choice was exercised by those in community‐based group home settings. This is in line with other studies that have shown the living environment is associated with the level of choice exercised by adults with intellectual disability (Cocks et al. 2017; Houseworth et al. 2018; O'Donovan et al. 2017; Robertson et al. 2019; Stancliffe et al. 2011; Wehmeyer and Bolding 2001). A multilevel modelling study by Houseworth et al. (2018) identified lower levels of choice were associated with more severe levels of intellectual disability and more problematic behaviours, while higher levels of choice were found in non‐agency settings and were associated with better mobility, ability to answer survey questions independently, and better verbal communication. All our study participants met criteria for having a mild to moderate intellectual disability and verbal communication skills, but it was not possible to discern if higher levels of disability and/or challenging behaviours were present in the group home settings.

Some of the limitations in choice and control noted in our sample associated with group home settings can be attributed to the presence of set routines and rosters, for example, cooking. Also, various choice items such as choosing and setting rules for the home, having a personal key to the home, attending day programs and having a say when selecting support staff were more restricted in these group home settings. This is again consistent with other studies that have found service‐related choices and environmental factors operate in these settings that are affected by disability policy, funding, regulations and service system support that include who to live with, having friends other than co‐residents, where to live, control over selection of staff, allocation of staff members and case manager, type of day/work activity undertaken and caregiver actions (Bigby et al. 2017; Heller et al. 1999; Houseworth et al. 2018; Lakin et al. 2008; O'Donovan et al. 2017; Sheppard‐Jones et al. 2005). The implications are that service providers, managers and staff of community‐based group home settings need to be mindful of these restrictions on individual choice and safeguard those they serve to avoid becoming institutionalised. This requires developing strategies and processes to maximise person‐centred practices and increase resident choice in these areas.

An important distinction between everyday choices and key life decisions was previously identified by O'Donovan et al. (2020) who explored choice opportunity and types among adults with intellectual disability over 40 years of age. Age did not emerge as a significant factor in our study although half our sample was aged 50 years and above. Nevertheless, our results indicated choice in key life decisions is not yet a reality. Little direct information was available on key life decisions among our participants, but there was evidence they had little choice about where they lived, and lower choice and control mean ratings were found on items that do have greater impact such as choosing to leave their job or day activity program, being able to have a pet, budgeting and choosing staff in a group home. Longer‐term or ‘key life decisions’ such as choosing where to live or work, who to live with, where to keep your money, exercising control over finances and choosing the amount and type of support received occur less often, but these decisions have much greater consequences than basic everyday choices such using the phone, choosing time to go to bed or how to spend free time. Risk factors and potential for greater consequences often influence caregivers and service providers to impose more restrictions around these key life choices (Houseworth et al. 2018; O'Donovan et al. 2017).

It is encouraging to see high levels of choice on everyday items, but there is clearly a need for greater attention to be given across all living environments to further promote and support choice‐making in key life areas, not just everyday choices. Evidence exists that choice‐making skills can be learned and/or improved by people with an intellectual disability (Harris 2003, 2016; Heller et al. 2011) and that such training can increase the opportunity for people with intellectual disabilities to speak up and communicate for themselves with or without assistance. As levels of choice increase, expectations of what choices are possible can also rise among people themselves and among family, caregivers and service providers alike. Williams and Porter (2017) also found self‐confidence to make one's own decisions, coupled with the support of trusted personnel who listen and respect the decisions made and enable risk taking, was critical for true choice and control among people with intellectual disabilities. Supported decision‐making training and resources are increasingly available and accessible via organisational websites such as the NDIS, National Disability Services (NDS; www.nds.org.au) and the Council for Intellectual Disability (CID 2024; https://cid.org.au/) providing training in choice‐making and supported decision‐making across key life areas is essential to increase choice and control across all QoL areas. Further investigation of the relationship between poor physical and/or mental health and wellbeing and level of choice is also recommended.

### 5.2 Wellbeing

Personal wellbeing scores indicated considerable variation in QoL being experienced among our NDIS participants. The distribution was positively skewed and results stable over the year of the study. The wellbeing average was similar to a population of people with mild to moderate intellectual disability well before the NDIS was introduced (McGillivray et al. 2009) and comparable or higher than the satisfaction levels previously reported for the general population (Capic et al. 2018; Cummins 2014; Cummins et al. 2004). The implication is that there has been no change in personal wellbeing domains represented here under the NDIS individualised funding model.

Poor physical health and/or poor mental and emotional health were identified as significant variables associated with greater dissatisfaction and lower scores on the personal wellbeing index. Poor physical and mental and emotional health was also negatively associated with lower levels of choice, and choice on items such as rulemaking and having a key to the home. Both physical and mental health were fundamentally associated with choice making and wellbeing.

Patterns across the seven PWB‐ID domains were akin to Cummin's study in which highest domain ratings were found for the ‘golden domain triangle’ of satisfaction with money, relationships and achieving in life, and lowest domain ratings being in health and future security (Cummins 2020). This is significant as Bertelli and Brown (2006) had noted that QoL and wellbeing and associated assessment instruments in the field of intellectual disability require further methodologically rigorous research, including longitudinal studies which examine daily practices and content effectiveness over time.

## 6 Limitations

Our sample was limited to people with mild to moderate intellectual disability who were verbal and could participate in the survey, so results may not be applicable to those with more severe or profound intellectual disability.

The Choice Questionnaire was chosen as a benchmark but there are obvious limitations in it. It does assess many everyday choices but needs to be extended to include not only basic everyday choices, but important key life decisions and service‐related choices. Some questions are relevant to group home situations but not to other residential settings such as the family home or living independently. Questions such as Who picks the staff to work in your house? Do you interview new staff to decide who will get the job? Are you asked what you think about new staff? are not relevant to people living at home with family and there is no guidance about how such questions should be scored. The scoring is also ambiguous regarding who makes the choice if someone lives at home with family. The person may still be making choices of what to do or someone else may be making a choice. It is time to update the scale to the new realities of where people may be living and clarify who makes the choice—the person independently, the person with support, the caregivers or service provider.

Further investigation of the relationship between poor physical and/or mental health and wellbeing and the level of choice is also recommended.

## 7 Conclusion

Physical health and mental and emotional health were identified as key variables influencing satisfaction as assessed by personal wellbeing, choice and NDIS individualised planning and support funding. Better physical health and/or mental emotional health resulted in higher satisfaction levels across all three of these dimensions, while poorer physical health and/or mental emotional health resulted in lower personal satisfaction, less choice and control and dissatisfaction with NDIS planning and support experiences for people with intellectual disability. While everyday choice and control is now being exercised, extremely limited choice and control is still apparent around key life choices. This is a critical area requiring greater focus, education and support across living environments, and especially in group home settings. Health and future security domains also warrant further attention to increase wellbeing and quality of life.

## Author Contributions

**Vivienne Riches:** conceptualisation, investigation, writing original draft. **Trevor Parmenter:** conceptualisation, investigation, writing original draft. **Gisselle Gallego:** project administrator, data curation, writing – review and editing. **Ziad Al‐Rubaie:** formal analysis, methodology. **Mary‐Ann O'Donovan:** conceptualisation, investigation, review, and editing. **Patricia O'Brien:** funding acquisition, conceptualisation, investigation, review and editing.

## Disclosure

The authors have nothing to report.

## Ethics Statement

Ethical approval was received by the Human research Ethics Committee of the University of Sydney 2021/047.

## Consent

Easy read versions of the participant information statement and consent forms were provided to the participants. All participants signed a consent form.

## Data Availability

Research data are not shared.
